# Plant-Mediated Horizontal Transmission of *Asaia* Between White-Backed Planthoppers, *Sogatella furcifera*

**DOI:** 10.3389/fmicb.2020.593485

**Published:** 2020-11-30

**Authors:** Fei Li, Hongxia Hua, Yongqiang Han, Maolin Hou

**Affiliations:** ^1^State Key Laboratory for Biology of Plant Diseases and Insect Pests, Institute of Plant Protection, Chinese Academy of Agricultural Sciences, Beijing, China; ^2^College of Plant Science and Technology, Huazhong Agricultural University, Wuhan, China; ^3^Hubei Biopesticide Engineering Research Center, Hubei Academy of Agricultural Sciences, Wuhan, China; ^4^College of Life Science and Environmental Resources, Yichun University, Yichun, China

**Keywords:** *Asaia*, *Sogatella furcifera*, plant-mediated, horizontal transmission, symbiont

## Abstract

*Asaia* is a bacterial symbiont of sugar-feeding insects that has been shown to be vertically transmitted by maternal transmission and paternal transmission mechanism, and to be horizontally transmitted via co-feeding artificial diet and venereal routes. Here, the first case of plant-mediated horizontal transmission of *Asaia* between white-backed planthoppers (WBPH), *Sogatella furcifera*, was reported. In *Asaia*-infected WBPH, *Asaia* was detected mostly in salivary glands and to a less extent in stylets. The rice leaf sheaths fed by *Asaia*-infected WBPH for 12 h were all positive with *Asaia*, where *Asaia* persisted for at least 30 d but was localized in the feeding sites only. When confined to *Asaia*-infected leaf sheaths for 7 d at the sites pre-infested by the *Asaia*-infected WBPH, all *Asaia*-free WBPH became infected with *Asaia* and the acquired *Asaia* could be vertically transmitted to their offspring. Phylogenetic analysis confirmed an identical *Asaia* strain in the *Asaia*-infected donor WBPH, the *Asaia*-infected leaf sheaths, and the newly infected recipient WBPH. Our findings provide direct evidence for the first time that rice plant can mediate horizontal transmission of *Asaia* between WBPH, which may contribute to the spread of *Asaia* in the field WBPH populations.

## Introduction

Most insect species harbor heritable symbionts. Recent studies estimate that about 2/3 of terrestrial arthropod species are infected with at least one species of heritable facultative symbiont (Jaenike, 2015). Given the increasing evidence of symbionts' functions in the interactions among host insects, their host plants and the environment (Oliver et al., 2010), it is crucial to know how the symbionts are transmitted.

Symbionts can be transmitted vertically and/or horizontally (Chiel et al., 2009; Chrostek et al., 2017). Vertical transmission occurs in many insect symbionts (Hosokawa et al., 2007), while horizontal transmission also exists in some symbionts (Oliver et al., 2010), which can be realized through parasitism, predation, mating, and feeding (Chiel et al., 2009; Gonella et al., 2015). The feeding route occurs in insects co-feeding on host plant, such as the transmission of *Hamiltonella defensa* in *Sitobion miscanthi* (Li et al., 2018), *Serratia symbiotica* in *Acyrthosiphon pisum* and *Aphis fabae* (Pons et al., 2019a; Skaljac et al., 2019), *Cardinium* in *Scaphoideus titanus* (Gonella et al., 2015), *Wolbachia* and *Rickettsia* in *Bemisia tabaci* (Caspi-Fluger et al., 2012; Li et al., 2017a,c), or on artificial diet (Crotti et al., 2009; Gonella et al., 2012, 2015), or on honeydew (Pons et al., 2019b).

*Asaia* is a bacterial symbiont associated with insects that feed on sugar-based diets (Crotti et al., 2010), particularly those in the order Diptera, Hemiptera, Hymenoptera, and Lepidoptera (Favia et al., 2007; Crotti et al., 2009; Li et al., 2017b; Ojha and Zhang, 2019; Zhang et al., 2019a,b). In addition to paternal transmission (Damiani et al., 2008), *Asaia* can be transmitted vertically via egg smearing (Crotti et al., 2009; Damiani et al., 2010). *Asaia* can also be horizontally transmitted through feeding route, such as between *S. titanus* and mosquitoes feeding on artificial diet mixed with *Asaia* cells (Crotti et al., 2009) and between *S. titanus* individuals through co-feeding artificial diet (Gonella et al., 2012), and through mating route (Damiani et al., 2008; Gonella et al., 2012). However, plant-mediated horizontal transmission of *Asaia* has not been observed.

The white-backed planthopper (WBPH), *Sogatella furcifera* (Hemiptera: Delphacidae), is one of the most destructive insect pests of rice in Asia (Fujita et al., 2013). Both the nymphs and adults co-feed gregariously at the basal parts of rice plants and cause damage by sucking phloem sap from rice leaf sheath (Rubia-Sanchez et al., 2003). During their feeding on rice plants, six distinctive waveforms have been recorded by electrical penetration graph (Lei et al., 2016). WBPH harbors a fungal yeast-like symbiont (Noda et al., 1995), and bacterial symbionts *Wolbachia* (Noda et al., 2001), *Cardinium* (Nakamura et al., 2012) and *Asaia* (Li et al., 2020). Specifically, *Asaia* has been revealed to play a role in improving WBPH fitness (Li et al., 2019). In a laboratory WBPH population, *Asaia* exists in all the individual WBPH, while *Asaia* is vertically transmitted at only 30% in WBPH (Li et al., 2019), indicating the potential for its horizontal transmission. However, there is no direct evidence showing plant-mediated horizontal transmission of *Asaia* between host insects including WBPH.

**Figure 1:**
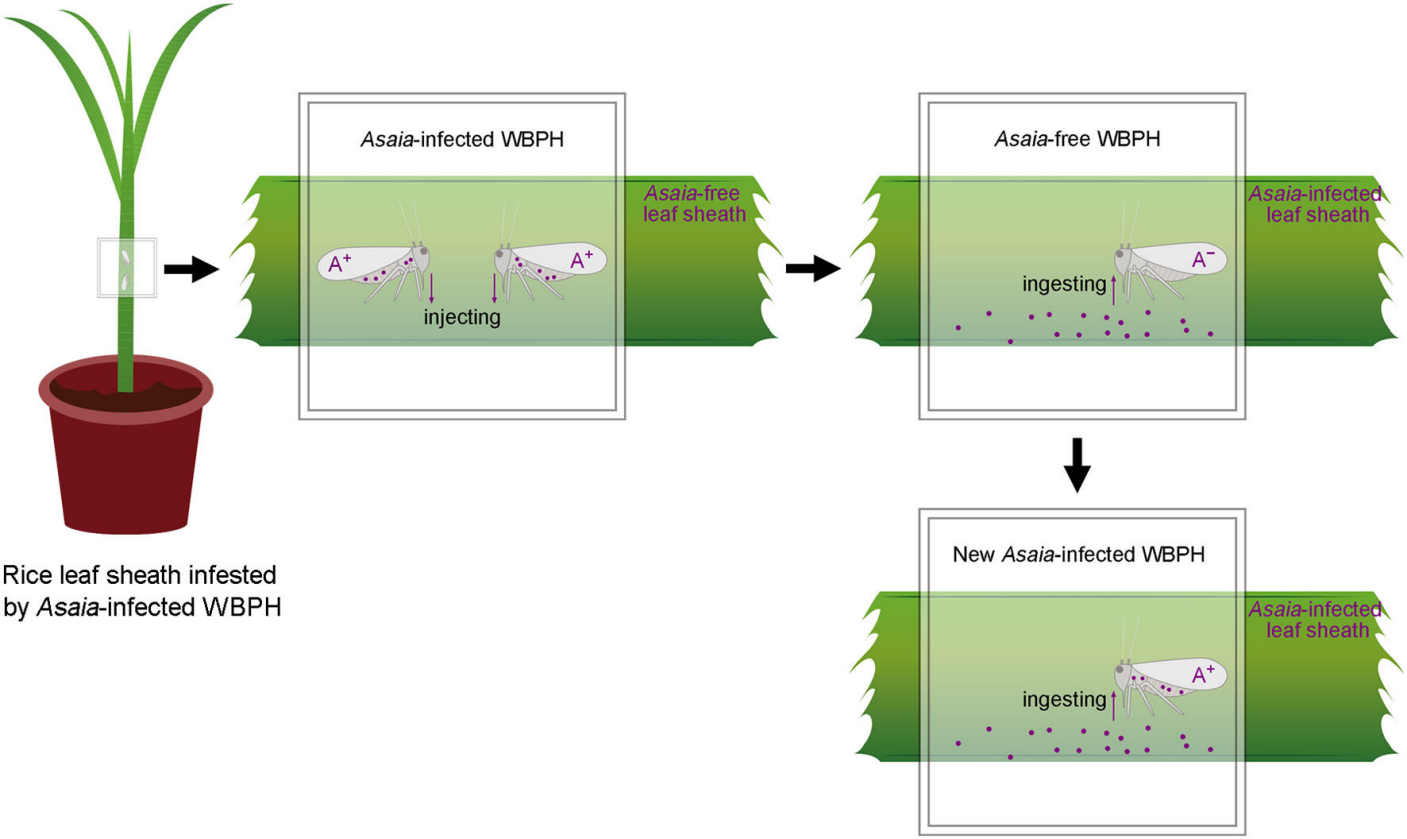
Schematic overview of the rice plant-mediated horizontal transmission of *Asaia*. A^+^, *Asaia*-infected WBPH; A^−^, *Asaia*-free WBPH; Dots, *Asaia*; Black frame, parafilm sachet.

The present study was designed to investigate if *Asaia* can be horizontally transmitted between WBPH via plants (Figure 1), i.e., transmission from *Asaia*-infected WBPH to rice leaf sheaths and subsequent acquisition by *Asaia*-free WBPH feeding on the *Asaia*-infected leaf sheaths. These questions were addressed through dynamic detection of *Asaia* in this plant-mediated transmission process by fluorescence *in situ* hybridization (FISH), diagnostic polymerase chain reaction (PCR), and quantitative real-time PCR (qPCR). Also, persistence and distribution of *Asaia* in the *Asaia*-infected rice leaf sheaths were determined.

## Materials and Methods

### Plants and WBPH Populations

Rice plants (var. Taichung Native 1, TN1) used in the experiments included tillering plants and 3-leaf plants. The tillering plants were soil-cultured from seedlings incubated in plastic plates (24 × 18 × 6 cm) containing organic soil (70% peat, 20% humus and 10% vermiculite) and then transplanted to plastic pots (18 cm in height and 18 cm in diameter) in 80-mesh cages in a greenhouse (30 ± 5°C, a photoperiod of 14 L: 10 D). The 3-leaf plants were hydroponically cultured from seeds placed in a glass tube lined with absorbent cotton at the bottom (18 cm in height and 3 cm in diameter, 15 seeds per tube), containing 20 ml rice nutrient solution, and sealed with insect-proof 80-mesh nettings in a phytotron (27 ± 1°C, relative humidity 80 ± 5% and a photoperiod of 14 L: 10 D).

The WBPH population (Lab population) was collected from rice fields in Xing'an (25°3601800 N, 110°4201600 E), China in 2014, and reared on caged rice seedlings in an insectary at the Chinese Academy of Agricultural Sciences (CAAS). Our previous study found that infection of *Asaia* in this population was 100% (Li et al., 2019). The Lab population served as an *Asaia* positive WBPH sub-colony. An *Asaia* negative WBPH sub-colony was established via oral treatment of the Lab population WBPH with tetracycline hydrochloride (Amresco, USA) as described by Li et al. (2019). The infection status of *Asaia* in these two sub-colonies was checked monthly against 20 randomly selected females and males using PCR with diagnostic primers Asafor and Asarev that amplify 16S rRNA gene (Favia et al., 2007) (Supplementary Figure 1).

### *Asaia* Transmission From WBPH to Rice Plants

To test transmission of *Asaia* from *Asaia*-infected WBPH to rice plants, a pair of newly emerged (<12 h) *Asaia*-infected WBPH adults, deprived of food for 1 h, were confined in a parafilm sachet (5 × 5 cm) attached to the leaf sheath of an *Asaia*-free tillering rice plant in a cage in the insectary and a total of 11 parafilm sachets were performed. At 0.5, 1, 2, 3, 4, 6, 8, 10, 12, 24 or 48 h post WBPH confinement in the sachet (11 treatments in total), the WBPH adults were removed and a 3-cm segment of the WBPH-confined leaf sheath part was collected. The experiment was repeated 30 times, and 30 leaf sheath segments were collected for each treatment. As a negative control, leaf sheath segments were collected from rice plants exposed to the *Asaia*-free WBPH adults in the same way. Because oviposition of WBPH begins 3.56 d post-emergence on average (Zhu and Cheng, 2001), transmission of *Asaia* to the rice sheaths via oviposition could be excluded.

Presence of *Asaia* in the WBPH-exposed leaf sheath segments was qualitatively detected using diagnostic PCR. To this end, total DNA was extracted individually from 20 leaf sheath segments randomly selected from each treatment using the Wizard® Genomic DNA Purification Kit (Promega, USA) according to the manufacturer's protocols. The *Asaia*-specific primers Asafor and Asarev were used to amplify a sequence of 181 bp of the 16S rRNA gene using 1 μl DNA extract for *Asaia* detection at conditions: 1 cycle of 94°C for 5 min; 35 cycles of 94°C for 30 s, 62°C for 30 s, and 72°C for 30 s; and a final extension of 72°C for 10 min (Favia et al., 2007). PCR amplified products were visualized on a 2% agarose gel containing GelRed colorant (Biotium, USA). If bands of the expected size were visible on the gels, the PCR products with the expected size were cloned into the pMD-19T plasmid vector (Takara, Japan) and were sequenced. Sterile water was included as a negative control and the DNA samples of WBPH verified by cloning and sequencing were used as a positive control in all PCRs.

*Asaia* densities in the WBPH-exposed leaf sheath segments were quantified by qPCR with the specific primers Asafor and Asarev using the remaining DNA extract of the 20 samples. To do this, five samples were randomly selected out from the 20 samples and pooled for the quantification. The quantification was performed in three biological repeats (15 samples used in total) each with three technical repetitions. qPCR reactions were conducted with SYBR® Premix Ex Taq™ II (Takara, Japan) in ABI 7500 Real-Time PCR System (Thermo Fisher Scientific, USA). The number of 16S rRNA gene copies of *Asaia* in the leaf sheath segments was calculated using absolute quantification analysis, following the protocol used in Li et al. (2019). Detailed procedures for qPCR *Asaia* detection are shown in Supplementary Method 1.

Presence of *Asaia* in the leaf sheaths and the *Asaia*-infected WBPH adults was also qualitatively detected using FISH. Twenty to thirty leaf sheath pieces (each 1.0 × 0.5 cm) were cut longitudinally from the rest of collected leaf sheath segments. Twenty to thirty WBPH salivary glands and heads were dissected from the donor *Asaia*-infected adults in a droplet of phosphate-buffered saline under a stereoscopic microscope. These samples were placed in Carnoy's solution and then hybridized with *Asaia*-specific Alexa Fluor 488-labeled 16S rDNA probe (A-488: 5′-GTGTAAACCGCCTACGCGCC-3′) (Damiani et al., 2010) using the method described by Li et al. (2020). The final samples were individually mounted on a slide with SlowFade antifade solution and observed under a laser scanning confocal microscope (Zeiss LSM 880, Carl Zeiss, Germany). *Asaia*-free WBPH and leaf sheaths fed by *Asaia*-free WBPH treated with the *Asaia* 16S rDNA probe were used as controls for confirmation of the specificity of *Asaia* detection.

### Persistence of *Asaia* in Rice Plants

To determine the persistence of *Asaia* in rice plants following its transmission from WBPH, a pair of newly emerged *Asaia*-infected WBPH adults starved for 1 h were confined to a leaf sheath in a parafilm sachet for 48 h as described above. This can ensure that *Asaia* have been transmitted from the WBPH to the plants because the above experiment showed that confinement of a pair of the *Asaia*-infected WBPH adults for 12 h resulted in *Asaia* transmission to the WBPH-confined leaf sheaths. A parafilm sachet without WBPH was used as a control. Upon removal of the sachet, the leaf sheath part attached with the sachet was labeled and a segment (~0.4 cm long) of the WBPH-confined leaf sheath part was cut for DNA extraction. This sample collection procedure was run every 5 d for a total period of 30 d. For each collection, three leaf sheath segments were collected. The collected leaf sheath segments were individually measured, each with three technical repetitions, for *Asaia* densities by qPCR using the specific primers Asafor and Asarev, following the protocol of Li et al. (2019).

### Distribution of *Asaia* in Rice Leaf Sheath

To examine the distribution of *Asaia* in the *Asaia*-infected leaf sheath, a test arena was designed, where a parafilm sachet containing a pair of newly emerged *Asaia*-infected WBPH adults was attached to the leaf sheath of an *Asaia*-free tillering plant and another parafilm sachet lacking WBPH was attached to the same leaf sheath about 3–5 cm below the sachet containing *Asaia*-infected WBPH adults. An *Asaia*-free tillering plant attached at the corresponding sites with a parafilm sachet containing a pair of *Asaia*-free WBPH and a blank sachet was included as the control. All the test rice plants were individually put in an insect-proof cage in the insectary. After 48 h, all the WBPH adults, along with their sachets, were removed. After another 5 d, a 3-cm segment of the WBPH-confined leaf sheath part was collected from each of the leaf sheaths attached with the sachets. The presence of *Asaia* in each leaf sheath segments was detected using diagnostic PCR with the specific primers Asafor and Asarev, using the method described in Favia et al. (2007). Three biological replicates were performed for each treatment.

### *Asaia* Transmission From Rice Plants to WBPH and Subsequent Vertical Transmission

To evaluate *Asaia* acquisition by WBPH from *Asaia*-infected rice plants, one newly emerged *Asaia*-free WBPH female adult was confined in a parafilm sachet attached to the feeding site of a pair of *Asaia*-infected WBPH on a tillering plant right after their 48 h infestation and removal from the leaf sheath. The Asaia-free WBPH females were left to feed on the rice sheaths for 1, 3, 5 or 7 d (the treatments) and a total of 30 females were tested in each treatment. *Asaia*-free WBPH females fed on *Asaia*-free leaf sheaths were used as a negative control. Twenty recipient females were randomly selected from each treatment for individual DNA extraction and 1 μl of the DNA extraction was used in detection of *Asaia* presence by diagnostic PCR with the specific primers Asafor and Asarev, using the method described in Favia et al. (2007). After diagnostic PCR, the remaining DNA of five individual WBPH females (five-WBPH-female DNA) in each treatment were randomly selected and pooled for quantification of *Asaia* using qPCR with the specific primers Asafor and Asarev, following the protocol of Li et al. (2019). The qPCR experiment was biologically repeated three times. The remaining 10 recipient WBPH females from each treatment were dissected for guts to examine *Asaia* presence using FISH.

Further tests were conducted to examine if the *Asaia* in WBPH acquired through feeding could be vertically transmitted to the next generation. A pair of the *Asaia*-free WBPH adults was fed on an *Asaia*-infected leaf sheath in a sachet for 7 d to ensure acquisition of *Asaia*. Then the pair of adults was introduced into a glass tube containing *Asaia*-free 3-leaf rice seedlings. After 24 h, the WBPH adults were removed for *Asaia* detection individually to confirm the infection of *Asaia* in the parent adults (F0 generation). If both parents were infected with *Asaia*, their newly hatched nymphs (F1 generation) were individually transferred to new glass tubes with *Asaia*-free 3-leaf rice seedlings and allowed to develop ad lib. Twenty females and males randomly selected from the resulting F1 adults were individually examined for the presence of *Asaia* by PCR with the specific primers Asafor and Asarev, using the method described in Favia et al. (2007). Three pairs of parental WBPH adults (F0) and their offspring (F1) were tested.

### Phylogenetic Analysis of *Asaia* in Relation With Transmission

A multilocus phylogenetic analysis was performed to conform the identity of the *Asaia* in the *Asaia*-infected donor WBPH, the *Asaia*-infected leaf sheath, and the newly infected recipient WBPH. Two *Asaia* genes: the 16S-23S rDNA internal transcribed spacer (ITS) and the *groEL* gene, were sequenced following the reported methods (Ruiz et al., 2000; Cleenwerck et al., 2010). The ITS and *groEL* gene sequences obtained in this study were registered with the GenBank database under accession numbers of MN095200-MN095202 and MN114618-MN114620, respectively. Multiple sequence alignments were performed using the program package Clustal W. The final alignments were manually inspected and corrected. Phylogenetic trees were constructed using the maximum likelihood (ML) method in MEGA v6.0 (Tamura et al., 2013). The ML trees were constructed with HKY + G model for ITS and K2 + I model for the *groEL* gene from *Asaia*. Bootstrap analysis of 1,000 replicates was used to deduce confidence levels.

## Results

### *Asaia* Transmission From WBPH to Rice Plants

**Figure 2:**
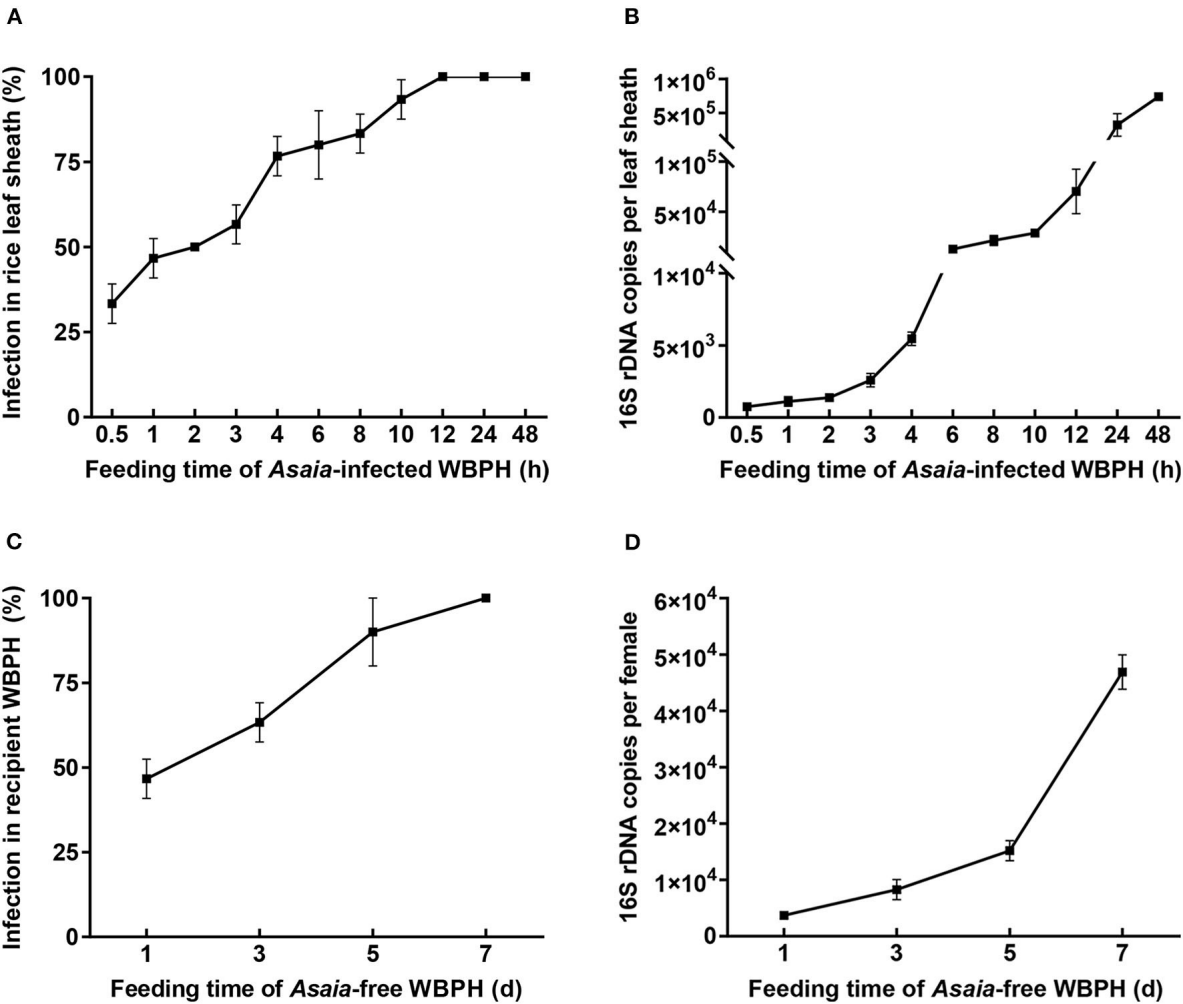
*Asaia* infection rate and density in leaf sheaths and recipient WBPH. **(A)** *Asaia* infection rate in leaf sheaths fed by *Asaia*-infected WBPH. **(B)** *Asaia* density in leaf sheaths fed by *Asaia*-infected WBPH. **(C)** *Asaia* infection rate in the recipient WBPH feeding on *Asaia*-infected rice leaf sheaths. **(D)** *Asaia* density in the recipient WBPH feeding on *Asaia*-infected rice leaf sheaths. The data are expressed as means ± sd.

When fed by the *Asaia*-infected WBPH, rice plants were infected with *Asaia* (Figures 2A,B; Supplementary Figure 1). Diagnostic PCR showed that the *Asaia* infection rate of rice plants showed a logistic pattern of increase with the extension of feeding by WBPH (Figure 2A). Feeding for 0.5 h by a pair of the *Asaia*-infected WBPH adults resulted in an infection rate of 33.3%. The infection rate reached 100% when WBPH feeding was 12 h or more. With qPCR, it is obvious that *Asaia* density in leaf sheaths also increased with the prolonged feeding by the *Asaia*-infected WBPH, in a pattern similar to an exponential increase (Figure 2B). During the initial feeding for 0.5–4 h, *Asaia* densities in leaf sheaths increased slowly, then increased sharply starting from feeding for 6 h.

**Figure 3:**
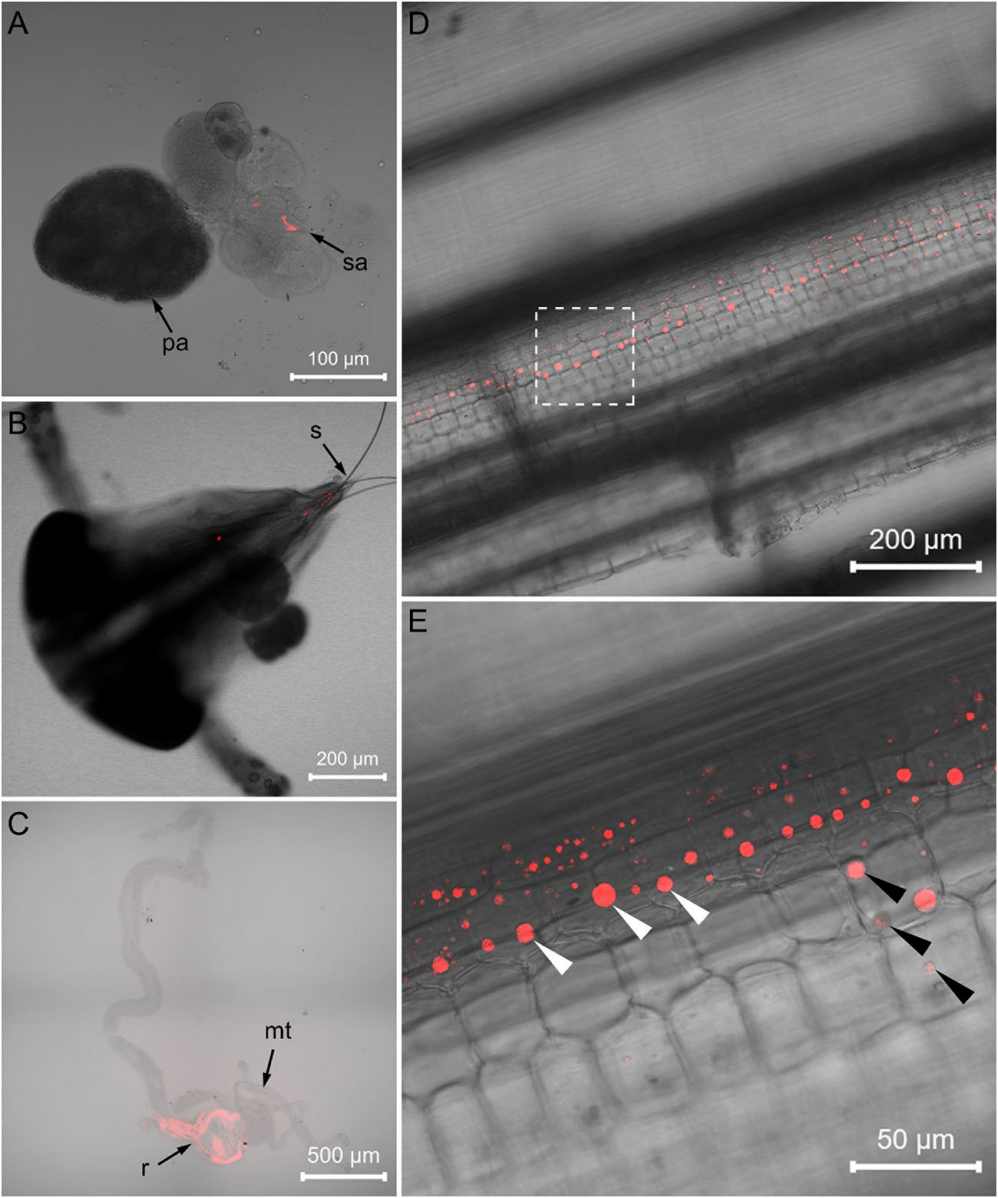
FISH visualization of *Asaia* in *Asaia*-infected WBPH and rice leaf sheath. **(A)** Salivary gland of the *Asaia*-infected WBPH. **(B)** Head of the *Asaia*-infected WBPH. **(C)** Infected rice leaf sheath. **(D)** Magnified image of **(C)**. **(E)** Gut of the newly infected recipient WBPH. pa, primary salivary gland; sa, accessory salivary gland; s, stylet; mt, Malpighian tubule; r, rectum; white arrowheads, *Asaia*; black arrowheads, sucked points.

FISH visualization showed that, in the *Asaia*-infected WBPH, masses of *Asaia* coalesced in the accessory salivary gland (Figure 3A) and a small amount of *Asaia* existed in the stylet (Figure 3B). In the leaf sheaths fed by the *Asaia*-infected WBPH, *Asaia* was distributed longitudinally along leaf vein in the sucked points (Figures 3D,E). No fluorescence of *Asaia* was visualized in the tissues of *Asaia*-free WBPH or in the leaf sheaths exposed to *Asaia*-free WBPH (Supplementary Figure 2).

### Persistence of *Asaia* in Rice Plants

**Figure 4:**
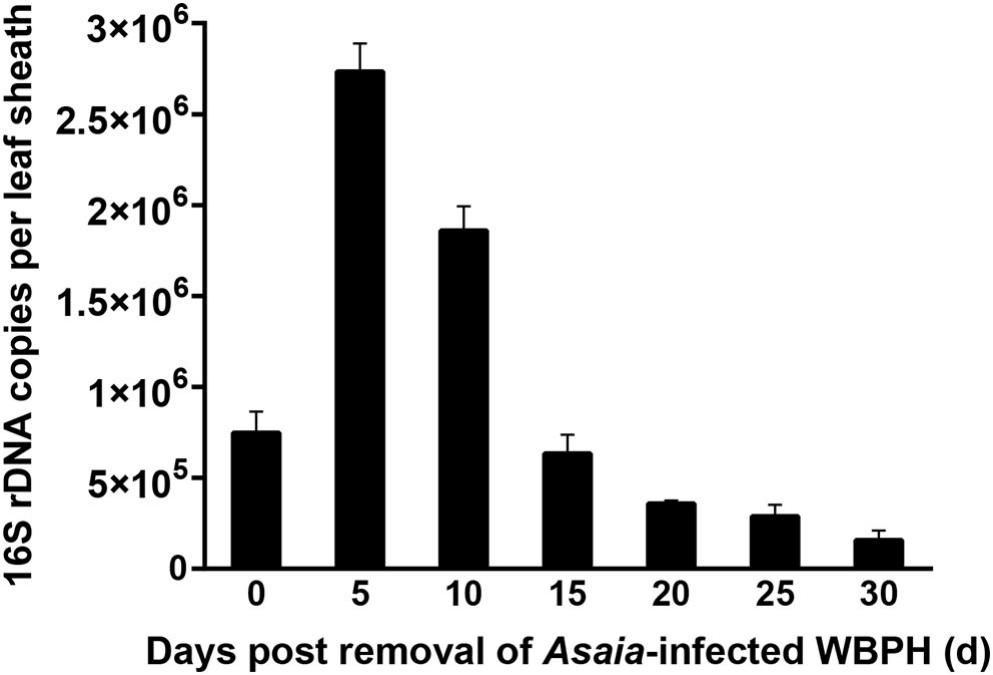
Persistence of *Asaia* in rice leaf sheaths horizontally transmitted from *Asaia*-infected WBPH through feeding. The data are expressed as means ± sd.

When rice leaf sheaths were exposed to a pair of *Asaia*-infected WBPH for only 48 h, *Asaia* densities in the leaf sheaths showed downward parabola dynamics during a 30-d period post the exposure (Figure 4). *Asaia* densities peaked at 5 d post exposure and then decreased. *Asaia* densities in the leaf sheaths at 30 d post exposure was reduced to 21.0% of the initial densities (at 0 d post exposure) and 5.8% of the peak densities (at 5 d post exposure).

### Distribution of *Asaia* in Rice Leaf Sheath

**Figure 5:**
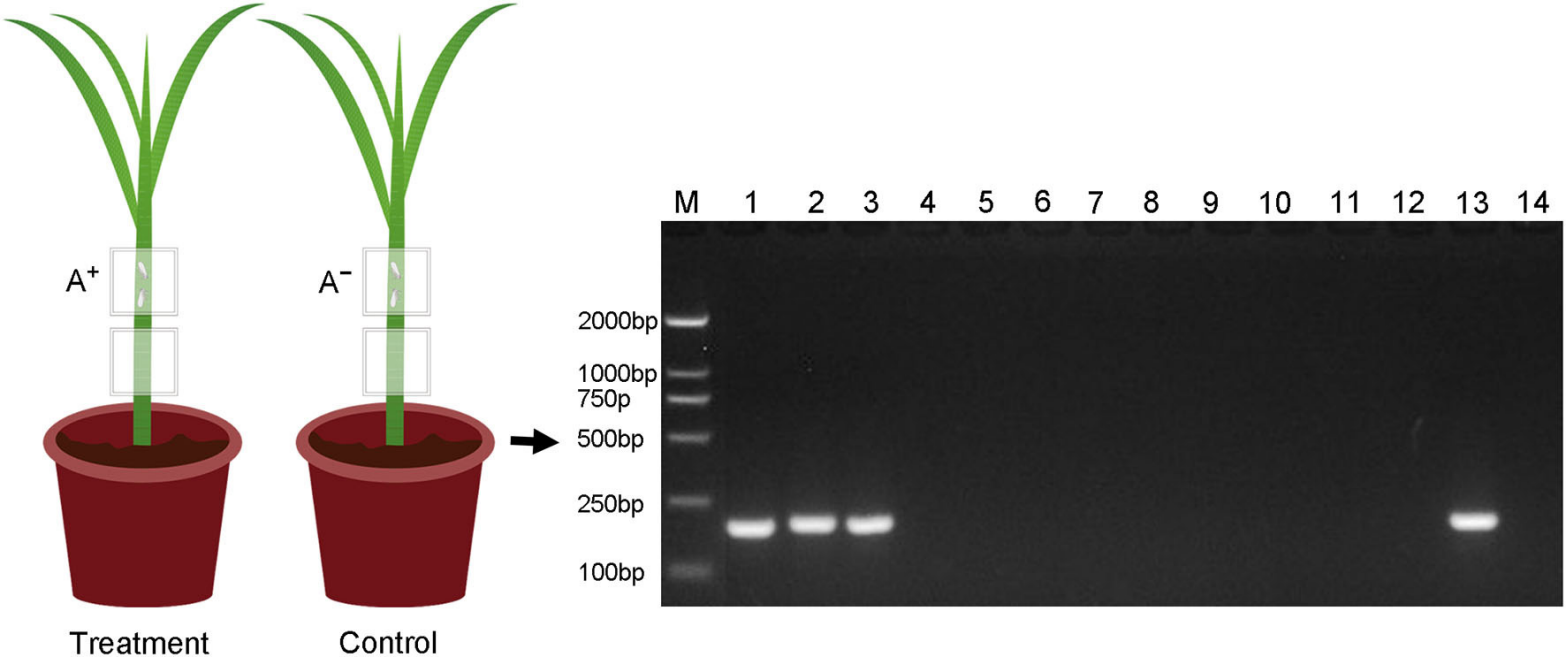
Detection of *Asaia* in different parts of the rice leaf sheaths fed by *Asaia*-infected and *Asaia*-free WBPH or not. M, DNA marker; Lanes 1–3, the upper leaf sheath segments fed by *Asaia*-infected WBPH (the treatment); lanes 4–6, the lower leaf sheath segments from the treatment without WBPH feeding; lanes 7–9, the upper leaf sheath segments fed by *Asaia*-free WBPH (the control); lanes 10–12, the lower leaf sheath segments from the control without WBPH feeding; lane 13, positive control of PCR; lane 14, negative control of PCR; A^+^, *Asaia*-infected WBPH; A^−^, *Asaia*-free WBPH.

*Asaia* in rice leaf sheath was restricted to the feeding sites, i.e., *Asaia* did not move in the leaf sheath. PCR detection showed that *Asaia* was detected only in the leaf sheath segments infested by the *Asaia*-infected WBPH, not from the leaf sheath segments below the feeding sites, and nor from any of the leaf sheaths in the control (Figure 5).

### *Asaia* Transmission From Rice Plants to WBPH and Subsequent Vertical Transmission

When the *Asaia*-free WBPH females were confined to the *Asaia*-infected rice sheaths, *Asaia* was successfully transmitted from the plants to the WBPH (Figures 2C,D; Supplementary Figure 1). The transmission was a feeding-time dependent process, with *Asaia* infection rates in the WBPH increasing almost linearly from 46.7% at feeding for 1 d to 100% at feeding for 7 d (Figure 2C). *Asaia* densities in the WBPH increased with the extension of feeding in an exponential manner (Figure 2D), being 12.6 times more at feeding for 7 d than at feeding for 1 d. FISH visualization showed heavy presence of *Asaia* in the rectum of the WBPH fed on *Asaia*-infected rice plants for 7 d (Figure 3C).

Vertical transmission of *Asaia* was measured in the WBPH that acquired *Asaia* from the *Asaia*-infected rice plants during a 7-d feeding period. The results showed that 40.0% of F1 female and 38.3% of F1 male WBPH adults were *Asaia* positive (Supplementary Figure 1). There was no significant sexual difference in the vertical transmission rate (*t* = 0.223, df = 4, *P* = 0.835).

### Phylogenetic Analysis of *Asaia* in Relation With Transmission

**Figure 6:**
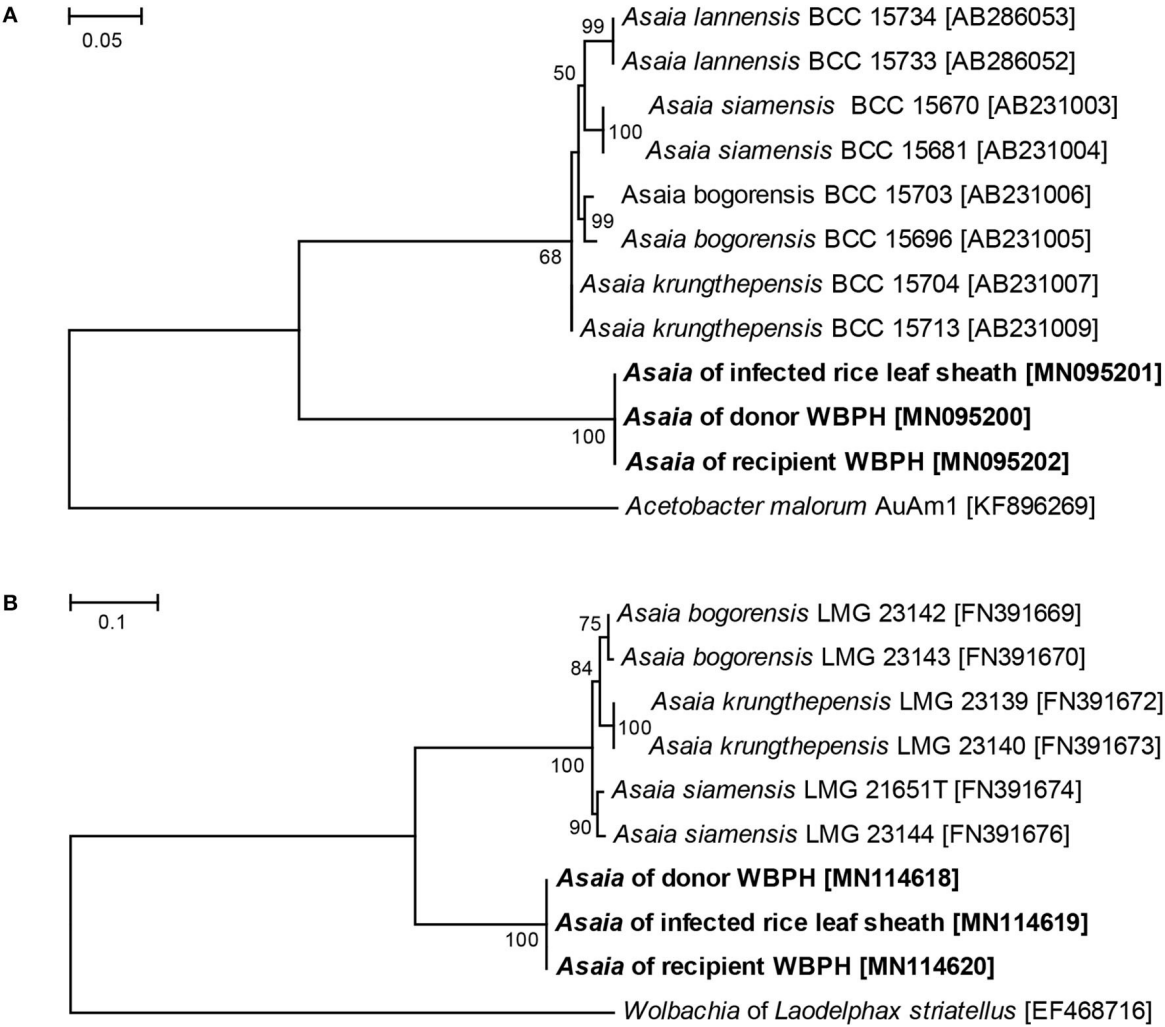
ML phylogenetic analysis of *Asaia* strains detected in different hosts. **(A)** ML tree based on *Asaia* ITS sequences. **(B)** ML tree based on *Asaia groEL* gene sequences. *Acetobacter malorum* AuAm1 and *Wolbachia* of *Laodelphax striatellus* were used as outgroups. Accession numbers of the sequences retrieved from GenBank are shown in square brackets. Sequences obtained in this study are shown in bold. The percent bootstrap values are shown at the nodes.

The *Asaia* sequences of ITS and *groEL* gene were used in the phylogenetic analysis. These genetic analyses revealed an identical *Asaia* strain in the *Asaia*-infected donor WBPH, the *Asaia*-infected leaf sheaths, and the newly infected recipient WBPH (Figure 6). These results show that the *Asaia* symbionts remain consistent during the plant-mediated horizontal transmission.

## Discussion

The horizontal transmission routes of symbionts have emerged as a focus of symbiont research over the past two decades (Moran and Dunbar, 2006; Jaenike et al., 2007; Caspi-Fluger et al., 2012; Ahmed et al., 2015; Li et al., 2017a,c, 2018; Pons et al., 2019a,b; Skaljac et al., 2019). Symbionts can be horizontally transmitted through such routes as parasitism, predation, mating, and feeding (Gonella et al., 2015). Previous studies have revealed that *Asaia* can be horizontally transmitted through co-feeding artificial diet (Crotti et al., 2009; Gonella et al., 2012) and through mating (Damiani et al., 2008; Gonella et al., 2012). Our current study provides evidence for the first time that *Asaia* can be horizontally transmitted via plant.

Symbionts colonize different organs in host insects, principally the salivary glands and guts, which are key organs involved in the horizontal transmission of symbionts (Chouaia et al., 2014). Our FISH visualization results showed *Asaia* in the salivary glands and stylets of the *Asaia*-infected donor WBPH (Figures 3A,B) and in the guts of the newly infected recipient WBPH (Figure 3C), which is consistent with *Asaia* localization in mosquitoes and leafhopper (Favia et al., 2007; Crotti et al., 2009; Gonella et al., 2012), and also in the leaf sheaths infested by the *Asaia*-infected donor WBPH (Figures 3D,E). Further, the *Asaia* in the newly infected recipient WBPH was vertically transmitted to their offspring at ca. 39%, corresponding to our previous detection of 30% heritable transmission (Li et al., 2019). In contrast, field WBPH populations are infected with *Asaia* at as high as 83.3% and in the laboratory population, at 100% (Li et al., 2020). These results indicate that horizontal transmission does occur in *Asaia*, especially in the case of laboratory population where the insects are confined in a cage. Furthermore, the phylogenetic analysis (Figure 6) revealed that the *Asaia* strain in the newly infected recipient WBPH is exactly the one from the *Asaia*-infected donor WBPH. Similar results were previously obtained with *Wolbachia* during horizontal transmission between two species of insect hosts (Ahmed et al., 2015; Li et al., 2017a). In addition, there is a possibility of transmission via honeydew. However, PCR revealed that the honeydews collected from the parafilm sachets with *Asaia*-infected WBPH were negative with *Asaia* (Supplementary Figure 1); and if honeydew contains *Asaia*, FISH will show massive fluorescence signals instead of dotted fluorescence signals as shown in this study (Figures 3D,E). Therefore, transmission via honeydew can be excluded. From these results, it is certain that plant-mediated horizontal transmission of *Asaia* does occur: *Asaia* harbored in the salivary glands of the *Asaia*-infected WBPH are injected with saliva via the stylet into the leaf sheaths, thus producing *Asaia*-infected rice plants; then the *Asaia* therein are ingested via the stylet into the gut of the recipient WBPH when they feed on the feeding sites of the *Asaia*-infected WBPH (Figure 1). The *Asaia* in the gut of the newly infected recipient WBPH finally cross the numerous physical and biochemical barriers to the salivary glands.

In this study, *Asaia* was quickly and efficiently transmitted from the *Asaia*-infected WBPH to rice plants. Akin to the transmission of a virus by virus-infected WBPH to rice plants (Lei et al., 2016), *Asaia* transmission is also a process that depends on the extent of feeding by the *Asaia*-infected WBPH (Figures 2A,B). Besides, symbiont transmission differs in their association with hosts. In contrast to the slow transmission of *H*. *defensa* by the aphids *S. miscanthi* (Li et al., 2018), *Asaia* transmission is quick; the difference may be that *Asaia* is an extracellular symbiont (Favia et al., 2007) while *H*. *defensa* is an extracellular and intracellular symbiont (Dykstra et al., 2014; Chrostek et al., 2017).

In the infected rice plants, *Asaia* persisted for as long as 30 d, like *Rickettsia* persisting in cotton leaves (Li et al., 2017c), although less than the 50-d persistence of *Wolbachia* in cotton leaves (Li et al., 2017a). The extended persistence may help enhance the chance of horizontal transmission of symbiont in the field. During the persistence of *Asaia* in the leaf sheaths, *Asaia* density experienced a downward parabola dynamic, peaking at 5 d post infestation by the *Asaia*-infected WBPH (Figure 4), indicating *Asaia* might have self-multiplied in plant tissues. It may be reasoned that the dynamic change of *Asaia* density in the leaf sheaths is a result of plant defense, in which the plants may take some time to mobilize certain chemicals to fight against or even eliminate the invading symbionts (Li et al., 2017c). However, this reasoning needs to be further investigated in future.

It is surprising that, in this study, *Asaia* couldn't be detected in the lower segment of the same leaf sheath that was infested with the *Asaia*-infected WBPH in an upper leaf sheath segment (Figure 5), indicating that *Asaia* is restricted locally to the WBPH feeding sites. This result is consistent with the localized infection of *H. defensa* in wheat leaves (Li et al., 2018), while contrary to that reported for *Rickettsia* (Caspi-Fluger et al., 2012; Li et al., 2017c), where *Rickettsia* move within the phloem and can be detected in the lower adjacent cotton leaves. The *Asaia*-free WBPHs were all infected with *Asaia* after feeding on *Asaia*-infected rice leaves for 7 d (Figure 2C), however this occurred in this study when they were confined to the feeding sites of the *Asaia*-infected WBPHs. The localized distribution of *Asaia* in the infected leaf sheath may limit its transmission, while this may be circumvented, to certain extent, by the efficient *Asaia* inoculation from the donor WBPH to rice plant, the extended *Asaia* persistence in rice plant, and the gregarious feeding habit of WBPH. The interconnection of these factors may enhance plant-mediated horizontal transmission of *Asaia* in nature.

Horizontal transmission may serve as a compensatory event to vertical transmission to rescue symbiont loss (Casiraghi et al., 2004) or replace the symbiont (Moran and Yun, 2015) in the host insect to help maintain the symbiont population in nature. In plant-mediated horizontal transmission, plants act as “springboards” for the symbionts to be spread to more host insects (Frago et al., 2012). Plant-mediated transmission of symbiont in WBPH has not been reported previously. Thus, the current results have broadened our understanding of symbiont transmission routes in WBPH. Whether horizontal transmission of *Asaia* occurs between *Asaia*-infected WBPH and other planthopper species needs to be investigated in the future, as *Asaia* has been detected in the small brown planthopper (*Laodelphax striatellus*) (Li et al., 2017b; Zhang et al., 2019b) and the brown planthopper (*N. lugens*) (Ojha and Zhang, 2019; Zhang et al., 2019a). Although most symbionts are transmitted vertically (Hosokawa et al., 2007), a recent study shows that, during plant-mediated horizontal transmission, symbionts orally secreted from the herbivorous hosts help suppress plant defenses (Moran and Yun, 2015). Thus, symbionts in herbivorous insects can not only confer numerous ecologically relevant traits to their hosts directly (Oliver et al., 2010), but also influence their hosts indirectly through involvement in insect-plant interactions (Beck et al., 2018) as a component of insect saliva (Zhang et al., 2017).

## Conclusions

Altogether, the current results have provided novel evidence showing the plant-mediated horizontal transmission of the bacterial symbiont *Asaia* between WBPH. The *Asaia* in the *Asaia*-infected donor WBPH is transmitted to rice plants quickly and efficiently when the insects feed on the leaf sheaths, and the *Asaia* in the infected leaf sheaths can also be efficiently acquired by the *Asaia*-free recipient WBPH when the insects feed on the leaf sheath sites pre-infested by the donor WBPH. The efficient *Asaia* inoculation from the donor WBPH to rice plants, the extended *Asaia* persistence in rice plants, and the gregarious feeding habit of WBPH can circumvent the limiting effects of the localized infection of *Asaia* in rice plants. Furthermore, the *Asaia* acquired by the *Asaia*-free recipient WBPH can be vertically transmitted to their offspring. Thus, the horizontal transmission via feeding route, coupled with vertical transmission, can help maintain *Asaia* in the field WBPH populations.

## Data Availability Statement

The datasets presented in this study can be found in online repositories. The names of the repository/repositories and accession number(s) can be found below: https://www.ncbi.nlm.nih.gov/genbank/, MN094401; https://www.ncbi.nlm.nih.gov/genbank/, MN094402; https://www.ncbi.nlm.nih.gov/genbank/, MN094403; https://www.ncbi.nlm.nih.gov/genbank/, MN095200; https://www.ncbi.nlm.nih.gov/genbank/, MN095201; https://www.ncbi.nlm.nih.gov/genbank/, MN095202; https://www.ncbi.nlm.nih.gov/genbank/, MN114618; https://www.ncbi.nlm.nih.gov/genbank/, MN114619; https://www.ncbi.nlm.nih.gov/genbank/, MN114620.

## Author Contributions

FL, HH, and MH conceived the idea, experimental design, and wrote the manuscript. FL and YH carried out the experiments. FL and MH analyzed the data. All authors contributed to the article and approved the submitted version.

## Conflict of Interest

The authors declare that the research was conducted in the absence of any commercial or financial relationships that could be construed as a potential conflict of interest.
