# Supplementary material for: Plant-Mediated Horizontal Transmission of Asaia Between White-Backed Planthoppers, Sogatella furcifera
Source: Front Microbiol. 2020 Nov 30;11:593485. doi: 10.3389/fmicb.2020.593485 (PMC7734105; doi:10.3389/fmicb.2020.593485)
Supplement: Supplementary file 2 [file Data_Sheet_2.zip › SUPPLEMENTARY FIGURE 1.docx]

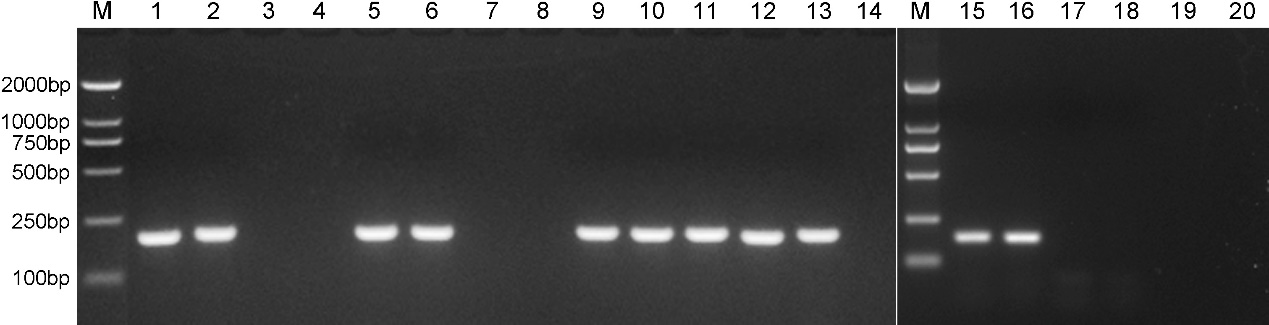


**SUPPLEMENTARY FIGURE 1 |** PCR detection of *Asaia* in WBPH, rice leaf sheaths and honeydew. M: DNA marker; Lanes 1−2: *Asaia*-infected WBPH; lanes 3−4: *Asaia*-free WBPH feeding on *Asaia*-free leaf sheath. lanes 5−6: *Asaia*-infected rice leaf sheath; lanes 7−8: *Asaia*-free rice leaf sheath; lanes 9−10: newly infected recipient WBPH; lanes 11−12: F1 offspring of the newly infected recipient WBPH; lanes 13, 15−16: positive control of PCR; lanes 14, 17: negative control of PCR; lanes 18−20: honeydew of *Asaia*-infected WBPH.
