# Supplementary material for: Plant-Mediated Horizontal Transmission of Asaia Between White-Backed Planthoppers, Sogatella furcifera
Source: Front Microbiol. 2020 Nov 30;11:593485. doi: 10.3389/fmicb.2020.593485 (PMC7734105; doi:10.3389/fmicb.2020.593485)
Supplement: Supplementary file 2 [file Data_Sheet_2.zip › SUPPLEMENTARY FIGURE 2.docx]

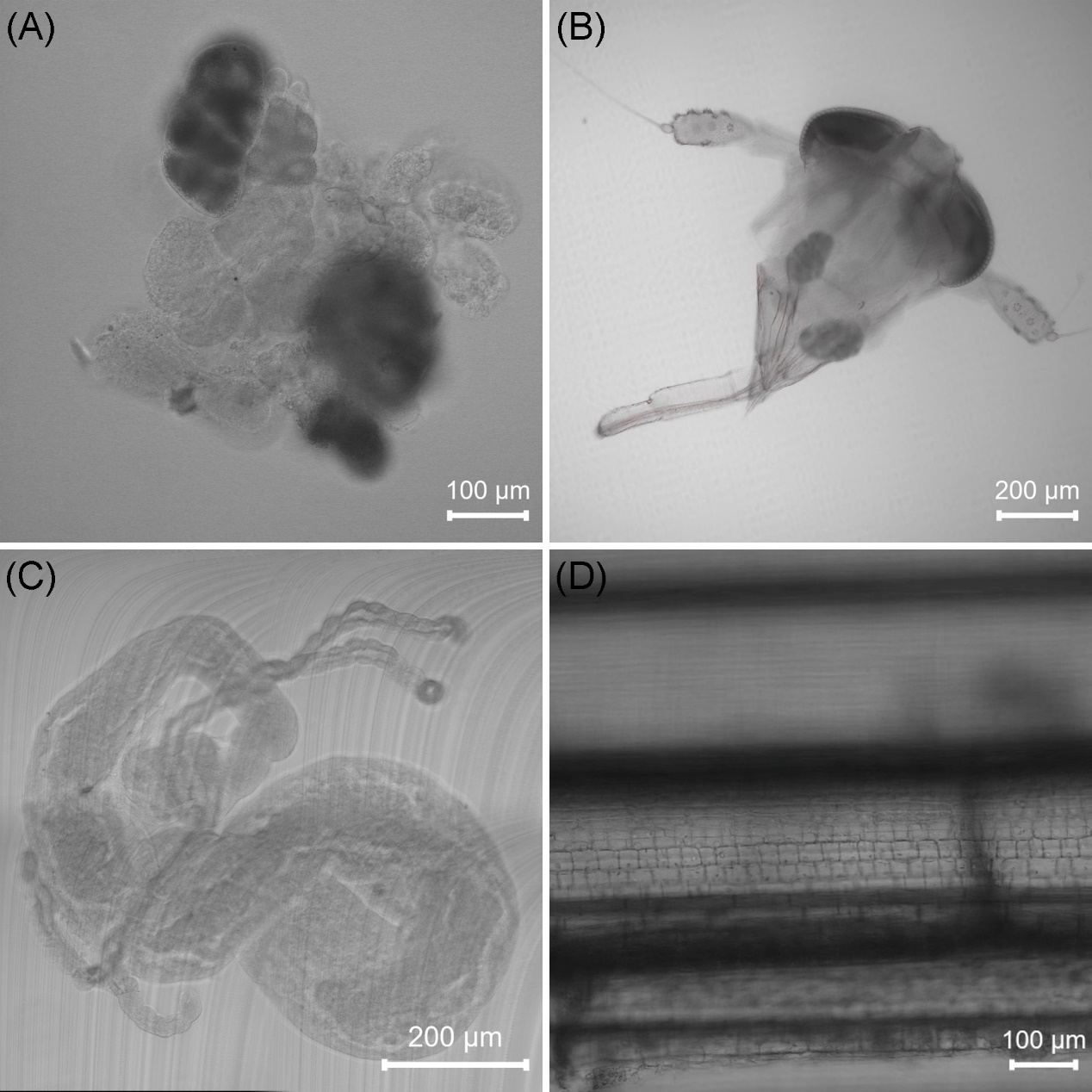


**SUPPLEMENTARY FIGURE 2 |** Negative controls of FISH visualization of *Asaia* in *Asaia*-free WBPH and rice leaf sheath fed by *Asaia*-free WBPH. **(A)** Salivary gland. **(B)** Head. **(C)** Gut. **(D)** Rice leaf sheath.
