# Supplementary material for: Plant-Mediated Horizontal Transmission of Asaia Between White-Backed Planthoppers, Sogatella furcifera
Source: Front Microbiol. 2020 Nov 30;11:593485. doi: 10.3389/fmicb.2020.593485 (PMC7734105; doi:10.3389/fmicb.2020.593485)
Supplement: Supplementary file 2 [file Data_Sheet_2.zip › SUPPLEMENTARY METHOD 1.docx]

**SUPPLEMENTARY METHOD 1.** The qPCR detection of *Asaia* in leaf sheath segments and WBPH females.

After diagnostic PCR, the remaining DNA (15 μl) of five individual leaf sheath segments (five-leaf-sheath DNA) or WBPH females (five-WBPH-female DNA) in each treatment were randomly selected and pooled. The quantitative detection of *Asaia* in leaf sheath segments and WBPH females by qPCR was performed in 20 μl reactions including 10 μl SYBR Premix Ex Taq II, 8 pmol of each primer, 2 μl pooled DNA and 6.4 μl ddH_2_O in ABI 7500 Real-Time System (Thermo Fisher Scientific, United States). The qPCR temperature profile was 2 min at 95^◦^C, 40 cycles of 5 s at 95^◦^C and 34 s at 60^◦^C. The primers used for qPCR detection were: Asafor: 5’-GCGCGTAGGCGGTTTACAC-3’ and Asarev: 5’-AGCGTCAGTAATGAGCCAGGTT-3’ (Favia et al., 2007). The qPCR reaction was biologically repeated in triplicate each with three technical replications. The number of 16S rRNA gene copies of *Asaia* in the leaf sheath segments and WBPH females was calculated using absolute quantification analysis. A serial of dilutions (10^2^, 10^3^, 10^4^, 10^5^, 10^6^, 10^7^, and 10^8^) of the standard plasmids, containing the sequence of 181 bp from the 16S rRNA gene of *Asaia*, was used as qPCR templates for the establishment of a standard curve: *y* = - 3.4486*x* + 37.97. The amplification efficiency with the primers was 95.0% for *Asaia*.
